# Combined vitamin D, ibuprofen and glutamic acid decarboxylase-alum treatment in recent onset Type I diabetes: lessons from the DIABGAD randomized pilot trial

**DOI:** 10.2144/fsoa-2020-0078

**Published:** 2020-06-23

**Authors:** Johnny Ludvigsson, Indusmita Routray, Sriramulu Elluru, Per Leanderson, Helena E Larsson, Björn Rathsman, Ragnar Hanås, Annelie Carlsson, Torben Ek, Ulf Samuelsson, Torun Torbjörnsdotter, Jan Åman, Eva Örtqvist, Karun Badwal, Craig Beam, Rosaura Casas

**Affiliations:** 1 Department of Biomedical & Clinical Sciences, Crown Princess Victoria Children´s Hospital & Div of Pediatrics, Linköping University, SE-58185, Linköping, Sweden; 2 Department of Biomedical & Clinical Sciences, Division of Pediatrics, Linköping University, SE 58185 Linköping, Sweden; 3 Department of Clinical & Experimental Medicine, Occupational & Environmental Medicine Center, Linköping University, Linköping S-58185, Sweden; 4 Pediatric Endocrinology, Department of Clinical Sciences Malmö, Lund University, Sweden & Department of Pediatrics, Skåne University Hospital, SE-21428 Malmö, Sweden; 5 Sachska Pediatric Hospital, Södersjukhuset, SE-11861 Stockholm, Sweden; 6 Department of Pediatrics, NU Hospital Group, SE 45153 Uddevalla, Sweden & Institute of Clinical Sciences, Sahlgrenska Academy at University of Gothenburg, SE 41346 Gothenburg, Sweden; 7 Pediatric Autoimmunity, Department of Clinical Sciences Lund, Lund University, Sweden, Skåne University Hospital, SE-22242 Lund, Sweden; 8 Department of Pediatrics, Hospital of Halland, SE 30233 Halmstad, Sweden; 9 Department of Women & Child Health, Astrid Lindgrens Children's Hospital at Karolinska University Hospital, Karolinska Institutet, SE 17164 Stockholm, Sweden; 10 Department of Pediatrics, University Hospital, SE 70382 Örebro, Sweden; 11 Department of Women & Child Health, Astrid Lindgren Children's Hospital at Karolinska University Hospital, Karolinska Institutet, SE 17164 Stockholm, Sweden; 12 Department of Internal Medicine, Western Michigan University Homer Stryker M.D. School of Medicine, Kalamazoo, MI, USA; 13 Department of Biomedical Sciences, Western Michigan University Homer Stryker M.D. School of Medicine, Kalamazoo, MI, USA

**Keywords:** C-peptide, GAD-alum, ibuprofen, immune response, Type I diabetes, vitamin D

## Abstract

**Aim:**

Double-blind placebo-controlled intervention using glutamic acid decarboxylase (GAD)-alum, vitamin D and Ibuprofen in recent onset Type I diabetes (T1D).

**Methods:**

64 patients (T1D since <4 months, age 10–17.99, fasting sC-peptide ≥0.12 nmol/l, GADA-positive) were randomized into Day(D) 1–90 400 mg/day Ibuprofen, D1–450 vitamin D 2000 IU/day, D15, 45 sc. 20 μg GAD-alum; as A but placebo instead of Ibuprofen; as B but 40 μg GAD-alum D15, 45; placebo.

**Results:**

Treatment was safe and tolerable. No C-peptide preservation was observed. We observed a linear correlation of baseline C-peptide, HbA1c and insulin/per kilogram/24 h with change in C-peptide AUC at 15 months (r = -0.776, p < 0.0001).

**Conclusion:**

Ibuprofen, vitamin D + GAD-alum did not preserve C-peptide. Treatment efficacy was influenced by baseline clinical and immunological factors and vitamin D concentration. Clinical Trial Registration: NCT01785108 (ClinicalTrials.gov).

## Lay abstract

In many countries, Type I diabetes with insufficient own insulin secretion is a common life-threatening disease in children and adults. There is no prevention and no cure. In spite of very intense treatment, the disease leads to serious complications. There is no efficaceous method to save own insulin secretion without serious risks and adverse events, but autoantigen treatment with glutamic acid decarboxylase has shown some efficacy. We have tried a combination therapy with vitamin D and anti-inflammatory treatment (ibuprofen). Vitamin D in combination with glutamic acid decarboxylase-alum seems to have beneficial effects, but not Ibuprofen. The effect is influenced by basal clinical and immunological status.

Type I diabetes is a chronic disorder requiring intensive lifelong treatment. Despite intensive treatment, this disease causes substantial morbidity and mortality [1,2]. Residual insulin secretion facilitates metabolic control, decreases the risk of keto-acidosis, and reduces the frequency of severe hypoglycemia episodes and long-term complications [3,4]. Several immune intervention regimens have shown some efficacy to preserve residual β-cell function [5–13], but usually the effect is transient, and in some cases associated with risks and adverse events. Administration of islet autoantigens seems to be safe and can be easily administered and tolerated without adverse effects. Treatment with glutamic acid decarboxylase (GAD)-alum showed some efficacy in a Phase II trial [14], but failed in a subsequent Phase II trial which used a different regimen and included a different age range of 3- to 45 year-old patients [15]. In a European Phase III trial, the treatment did not reach the primary end point, but efficacy was found in some pre-specified subgroups such as in males, in non-nordic countries and in individuals with moderate HLA risk [16]. It cannot be excluded that the ‘swine flu’ pandemic and accompanying mass vaccination might have played a role [17,18]. A meta-analysis using Bayesian methods showed that the probability of GAD-alum preserving residual β-cell function is 97% [19]. Thus, treatment with GAD-alum might be beneficial for some patients but has not been sufficiently effective. One possibility to improve efficacy would be to select the most suitable patients. The previous Phase III trial showed the best (and statistically significant) results in boys [16], but we did not feel prepared to select only boys. As we believe that the efficacy earlier seen in non-Nordic countries [16] might be explained by the swine-flu vaccination [17,18] we dared to include Swedish patients but decided not to allow any other vaccination close to the GAD-alum treatment. As patients in the Phase III trial with extremely-high-GADA titers (>50,000 U/ml) failed to respond to the GAD-alum treatment [16] we drew the conclusion not to include patients with GADA >50,000 U/ml at baseline. On the other hand, patients should have clear GADA positivity as patients with very low GADA (<25 U/ml) in the Phase III trial did not respond.

In addition to these conclusions drawn from previous studies, we looked for other ways to improve efficacy.

Vitamin D is thought to have multiple positive benefits, including improving dendritic cell function, as vitamin D is able to inhibit DC differentiation and maturation into APCs, therefore rendering DC cells more tolerogenic [20]. Vitamin D can also induce TH2 deviation [21], and it seems to inhibit surface expression of MHC class II and co-signaling molecules on antigen presenting cells, reduce the activity of Th1 and Th17 cells and upregulate regulatory T cells (Tregs), thus leading to a shift of T cells from an effector phenotype to a regulatory phenotype [22]. Furthermore, vitamin D may protect β cells and improve insulin sensitivity [23,24]. Its contributing role in the pathogenesis of Type I diabetes is supported by epidemiological studies demonstrating higher incidence of Type I diabetes in northern latitudes where a decreased exposure to sunlight results in decreased endogenous synthesis of vitamin D [25]. Consequently, this has become an area of research with multiple studies investigating the role of vitamin D supplementation in the preservation of β-cell function. However, the effects of vitamin D alone on preserving β-cell function were transient in these studies [26,27]. Based on those studies, we chose a dose of 2000 U/day of cholecalciferol, supposed to be enough for efficacy but still safe, as it is far below the doses, up to 4000 U/day, supposed to be safe for children in this age group [28].

Although Type I diabetes is regarded as an autoimmune disease, there are several studies both in experimental animals and in humans suggesting that inflammation plays an important role [29]. Type I diabetes has been shown to be associated with increased cyclooxygenase- and cytokine-mediated inflammation [30]. Ibuprofen, which mainly blocks cox-2 but to some extent also cox-1 [31], has a quite good anti-inflammatory effect without showing serious risks or adverse events. It is commonly used even in children and regarded as a very safe drug. We chose 400 mg/day as a common dose for children, safe and still with well-known effect.

In a recent review addressing the use of auto-antigen therapies in Type I diabetes [32], a group of experts in the field has highlighted the relevance of trying different combination therapies to understand more about mechanisms and future selection of subgroups of patients [33]. Based on the same opinion [34], we have consciously conducted a series of small pilot experiments in the last few years. Thus, here we report results from the DIABGAD study where we sought to improve efficacy of GAD-alum by combining it with vitamin D and with ibuprofen. We had the following hypotheses: administration of GAD-alum twice (at day 15 and 45; the 1-month interval was chosen in agreement with previous GAD-alum studies) may downregulate the autoimmune process and contribute to the preservation of residual insulin secretion; as previous studies have indicated, the dose should be somewhere between 20 and 100 μg GAD-alum, and a higher dose than used in previous studies, 40 μg GAD-alum per occasion, may augment the preservation effect. Addition of rather large doses of vitamin D supplement may enhance the efficacy by exerting its effect on the immune system and directly on the β cells; treatment with ibuprofen, before and/or during treatment with GAD-alum, may decrease the ongoing inflammation and improve/maintain the effect of GAD-alum treatment enforced by vitamin D supplementation.

We aimed to explore the clinical and immunological mechanisms and possible efficacy of some treatment arms. Several other treatment arms would have been interesting and could have given more answers, but we had to restrict the size of the study for practical reasons and based on resources.

The study (ClinicalTrials.gov: NCT01785108) was approved by the Medical Product Agency in Sweden and by the Research Ethics Committee, Linköping (Dnr 2012/73-31). All patients and their parents/caregivers gave their consent after oral and written information.

## Patients & design

The study was a multicenter, randomized, four-arm, double-blind, placebo-controlled clinical trial. A formal sample size calculation was not performed for this pilot study, which aimed to include 60 patients; we are aware of the low power to show significant differences in efficacy. The patients were selected based on the following inclusion criteria: Type I diabetes according to the ADA classification with <4 months diabetes duration at the time of screening, age 10.00–17.99 years at time of screening, fasting serum C-peptide at the time of screening ≥0.12 nmol/l and positive for GAD65-autoantibodies (GADA) but <50,000 U/ml. Out of 78 patients screened at nine Swedish pediatric clinics, 64 were eligible for the study. Baseline characteristics of the study subjects are summarized in Table 1. Participants were randomized into one of four groups: patients (n = 16) were assigned to receive 400 mg Ibuprofen (in solution) day 1–90, and from day 1 to 450 they also received oral vitamin D (cholecalciferol) 2000 IU drops daily. In addition, two injections, one sc. injection with 20 μg GAD-alum (Diamyd™) plus one with placebo, on days 15 and 45, in other words, prime and booster dose. GAD-alum sc. injections were administrated in the stomach area in close proximity to the pancreas and its neighboring lymph nodes to attempt to augment the effect. Patients (n = 16) were assigned to receive placebo (in solution) day 1–90, and from day 1 to 450 they received daily oral vitamin D 2000 IU drops. In addition, they were given two injections, one sc. injection with 20 μg GAD-alum (Diamyd) and one with placebo, on days 15 and 45. Patients (n = 16) were assigned to receive placebo (in solution) day 1–90, and from day 1 to 450 they received daily oral vitamin D 2000 IU drops. In addition, they received two sc. injections with each 20 μg GAD-alum (Diamyd™) at two different sites, that is altogether 40 μg GAD-alum (Diamyd) on day 15 and 45. Patients (n = 16) were assigned to receive placebo solution day 1–90, and then oral placebo oral drops from day 1 to 450, and two placebo sc. injections on days 15 and 45.

**Table 1.** Baseline patient characteristics: sex, age, weight, height and diabetes-related parameters (total population).

Variable	Response category	Group A (n = 16)	Group B (n = 16)	Group C (n = 16)	Group D (n = 16)
Gender	Females	6	7	10	9
	Males	10	9	6	7
Age (years)	Mean (SD)	13.3 (1.9)	14.4 (2.7)	13.3 (2.4)	14.2 (2.2)
	Median	13.6	15.1	130.0	14.4
	Min	10.0	10.2	10.1	10.2
	Max	15.5	17.8	17.8	180.0
T1D duration (days)	Mean (SD)	94 (34)	88 (35)	97 (31)	74 (34)
	Median	83	77	95	70
	Min	53	35	28	18
	Max	143	138	138	138
Weight	Mean (SD)	52.0 (12.6)	530.0 (15.8)	47.9 (15.0)	56.5 (13.5)
	Median	52.6	50.8	46.2	560.0
	Min	32.6	29.9	29.4	28.0
	Max	71.2	83.7	73.2	80.7
Height	Mean (SD)	162.6 (13.8)	163.2 (13.7)	158.2 (15.4)	163.5 (12.8)
	Median	163.4	164.8	159.8	161.2
	Min	135.5	138.8	138.9	1320.0
	Max	183.1	188.0	183.7	1870.0
BMI	Mean (SD)	19.2 (2.4)	19.8 (4.2)	19.0 (2.6)	21.1 (2.8)
	Median	19.5	18.7	18.4	21.4
	Min	15.8	15.2	15.1	15.9
	Max	22.8	27.9	23.8	25.7
IA-2A (U/ml)	Mean (SD)	2710 (2920)	2988 (6176)	3535 (4477)	930.8 (804.5)
	Median	1638	1129	1374	1122
	Min	38.5	4.7	11.8	5.3
	Max	8390	23300	14050	2410
Fasting glucose (mmol/l)	Mean (SD)	6.5 (20.0)	6.4 (1.9)	6.6 (1.7)	5.7 (1.2)
	Median	6.2	6.0	6.8	5.6
	Min	4.6	3.6	4.2	3.7
	Max	12.8	12.4	10.3	7.5
HbA1c (mmol/mol)	Mean (SD)	43.69 (7.39)	49.56 (9.83)	46.88 (6.44)	46.94 (9.83)
	Median	43.50	47.50	470.00	44.50
	Min	32.00	330.00	33.00	370.00
	Max	59.00	680.00	58.00	770.00
Average insulin dose/kg/24 h (IU)	Mean (SD)	0.521 (0.2)	0.615 (0.301)	0.488 (0.225)	0.596 (0.402)
	Median	0.506	0.592	0.540	0.522
	Min	0.300	0.089	00.077	0.161
	Max	1.125	1.359	1.036	1.807
Fasting C-peptide (nmol/l)	Mean (SD)	0.312 (0.186)	0.244 (0.137)	0.301 (0.123)	0.238 (0.119)
	Median	0.300	0.210	0.250	0.225
	Min	00.080	0.080	0.180	00.040
	Max	0.800	0.590	0.620	0.430
GADA (U/ml)	Mean (SD)	580 (876)	1111 (1491)	4309 (6479)	1901 (4161)
	Median	317	570	1308	302
	Min	60	70	135	59
	Max	3370	4825	19,950	15,590
C-peptide AUC/120 min (nmol/l/min)	Mean (SD)	0.770 (0.339)	0.608 (0.202)	0.680 (0.281)	0.666 (0.213)
	Median	0.693	0.645	0.644	0.716
	Min	0.339	0.221	0.364	0.196
	Max	1.313	0.971	1.338	0.953
Vitamin D (nmol/l)	Mean (SD)	48.4 (16.6)	54.6 (20.6)	58.4 (12.7)	54.1 (9.4)
	Median	48.1	51.2	60.5	54
	Min	17.7	15.3	38.5	29.9
	Max	73.8	99.7	83.6	72.1

**Figure 1.**
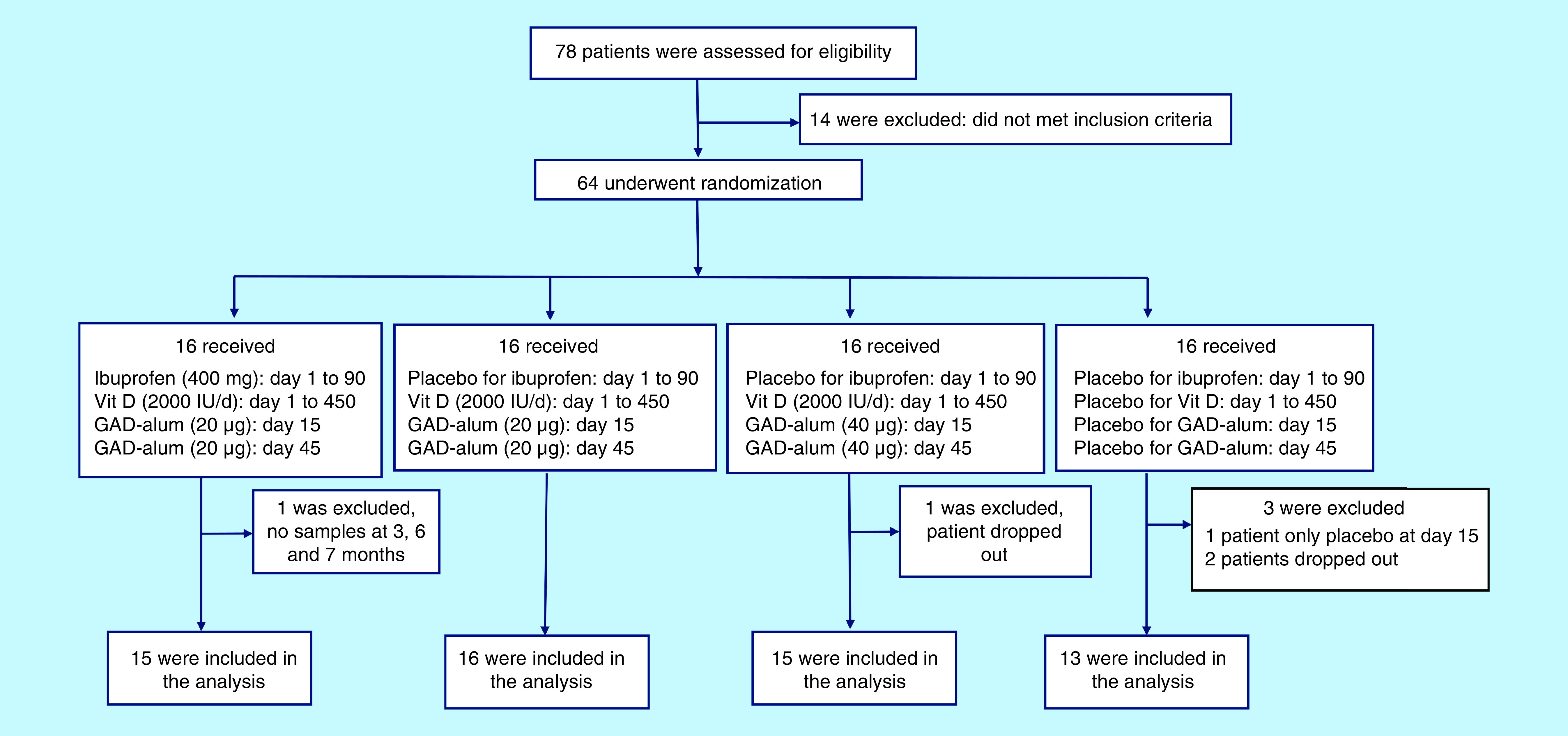
Flow chart showing the recruitment and distribution of patients into the different groups.

The patients were recruited between 2013 and 2015 and followed for a total duration of 30 months, blinded as to the clinicians, patients and parents. Over the duration of the study, three patients dropped out (one because of adverse event [nausea] in the placebo group, and two who did not want to continue), several samples were lacking in one patient and one patient violated the protocol. As a result, 64 patients were in the Intention To Treat (ITT) group and 59 patients in the Per Protocol Set (PPS) at 30 months. Only 49 patients were eligible for comparison at baseline and 30 months as some samples were missing (Figure 1).

The first objective of the trial was to evaluate the safety of the different treatment arms and then the effect on immune system and preservation of residual β-cell function. No specific primary end point was specified, but efficacy end points were a change in C-peptide, both the 90-min value and the AUC/120 min (Area Under Curve during the 120 min long mixed meal tolerance test [MMTT]) from baseline to month 15 and to month 30, respectively, as well as fasting C-peptide. We also compared the proportion of patients with a maximum stimulated C-peptide above 0.2 nmol/l, at baseline and after 6, 15 and 30 months. We also wanted to investigate the effect on HbA1c and insulin dose.

The safety assessment included routine vital sign and laboratory measurements, a regular observation of the injection sites for reactions and the occurrence of adverse events (AEs).

## Laboratory tests

Laboratory analyses were performed at Linköping University, Sweden. Blood samples were drawn after fasting overnight. Venous blood from the cubital vein was collected into sodium-heparinized tubes for PBMC separation. Serum gel tubes were used for analysis of autoantibodies and C-peptide and EDTA tubes for the measurement of HbA1c.

Analysis of serum C-peptide was performed using a solid-phase, two-sided enzyme immunoassay (Mercodia, Uppsala, Sweden). Results for each assay were validated with the inclusion of a Diabetes Antigen Control Human (low/high) (Mercodia). The assay was calibrated against the International reference reagent for C-peptide IRR c-peptide 84/510. Inter- and intra-assay variation were 6.6 and 3.5%, respectively.

Serum GAD65 autoantibodies (GADA) were estimated in duplicate with a radio-binding assay using ^35^S-labeled recombinant human GAD_65_ as previously described [26]. Sepharose protein A was used to separate free from antibody-bound labeled GAD_65_. A diabetes autoantibody standardization program (IASP) in which the laboratory participated has shown that GADA assay has a sensitivity of 70% and specificity of 100%.

Vitamin D (25-hydroxyvitanin D2 and D3) was analyzed with high-performance liquid chromatography-electrospray tandem mass spectrometry after plasma extraction and derivatization with PTAD (4-phenyl-1,2,4-triazoline-3,5-dione). Quality of the assay was assured by participation in the Vitamin D External Quality Assessment Scheme (DEQAS). Reference standards from DEQAS were also analyzed together with the samples.

Lymphocyte proliferation and cytokine secretion assays were performed in samples from baseline and 15, 45, 90 and 180 days. We studied the PBMC proliferative responses in the presence of 5 μg/ml rhGAD65 (Diamyd Medical, Stockholm, Sweden), CD3/CD28 beads (Gibco, Life Technologies AS, Oslo, Norway), PMA/ionomycin (Sigma) and in medium alone. Data were expressed as stimulation index, calculated as the mean of triplicates in presence of stimulus divided by the mean of triplicates with medium alone. Cytokines were quantified both in serum samples and in peripheral blood mononuclear cells (PBMCs) supernatants. PBMC were cultured for 7 days in the presence of 5 μg/ml recombinant human GAD65 (Diamyd Medical, Stockholm, Sweden) or in medium (AIM-V) alone at 37°C in 5% CO2. The cytokines interleukin IL-1, IL-2, IL-4, IL-5, IL-10, IL-13, IL-17, TNF-α and IFN-γ were using Bio-Plex Pro Cytokine Panel (Bio-Rad, CA, USA) according to the manufacturer's instructions. The chemokines Eotaxin, IL-10, MCP-1, MIP-a, MIP-b and IL-8 were quantified in serum samples. Data was collected using the Luminex 200™ (Luminex xMAP™ Corporation, TX, USA). The antigen-induced cytokine secretion level was calculated by subtracting the spontaneous secretion (i.e., secretion from PBMC cultured in medium alone) from the one following stimulation with GAD65.

## Statistics

Demographics and baseline characteristics are presented using descriptive statistics. Efficacy data regarding C-peptide and immune system, as well as Serious (S) Adverse Events (AE) and other safety data are also summarized descriptively. The AE/SAE are presented using a standardized tabulation of the frequency and incidence rate of all observed AEs/SAEs. The frequencies and incidence rates are calculated on a per-patient basis. Mixed-effects generalized linear modeling was conducted using SAS (v9.4) to determine the longitudinal effects of treatment. These models include random effects for subject in order to account for longitudinal (‘within subject’) correlation. A series of pre-planned contrasts were used to compare treatment groups with respect to various changes in the primary variables from one point in time (baseline, month 6, month 15) to another (month 6, month 15 and month 30). No adjustment for multiple testing was used in the analysis of this pilot study. Prior to statistical modeling itself, transformations of the data (e.g., logarithmic) were investigated for variance reduction and normality. p-values <0.05 were considered statistically significant.

Canonical correlation analysis (CCA) was used to determine a combination of metabolic factors most strongly correlated to C-peptide change from baseline. CCA is similar to principal components analysis (PCA) in that they both reduce the dimensionality of the data to a smaller number of independent components. However, whereas the components determined in PCA are meant to explain the greatest amount of variation in the data, the components in CCA are meant to have the greatest correlation with a predicted variable (e.g., C-peptide).

## Results

There were no serious adverse events and no significant differences in adverse events between the different groups, except for an increase of transient mild reactions at the GAD-alum injection sites in the active arms compared with placebo (Supplementary Tables 2 & 3).

No statistically significant differences between treatment groups were found in C-peptide, insulin dose or HbA1c changes or the combination of insulin dose and HbA1c, IDAAC [35] from baseline (visit 2, start of study) to 30 months, with no difference related to sex or ethnicity.

**Figure 2.**
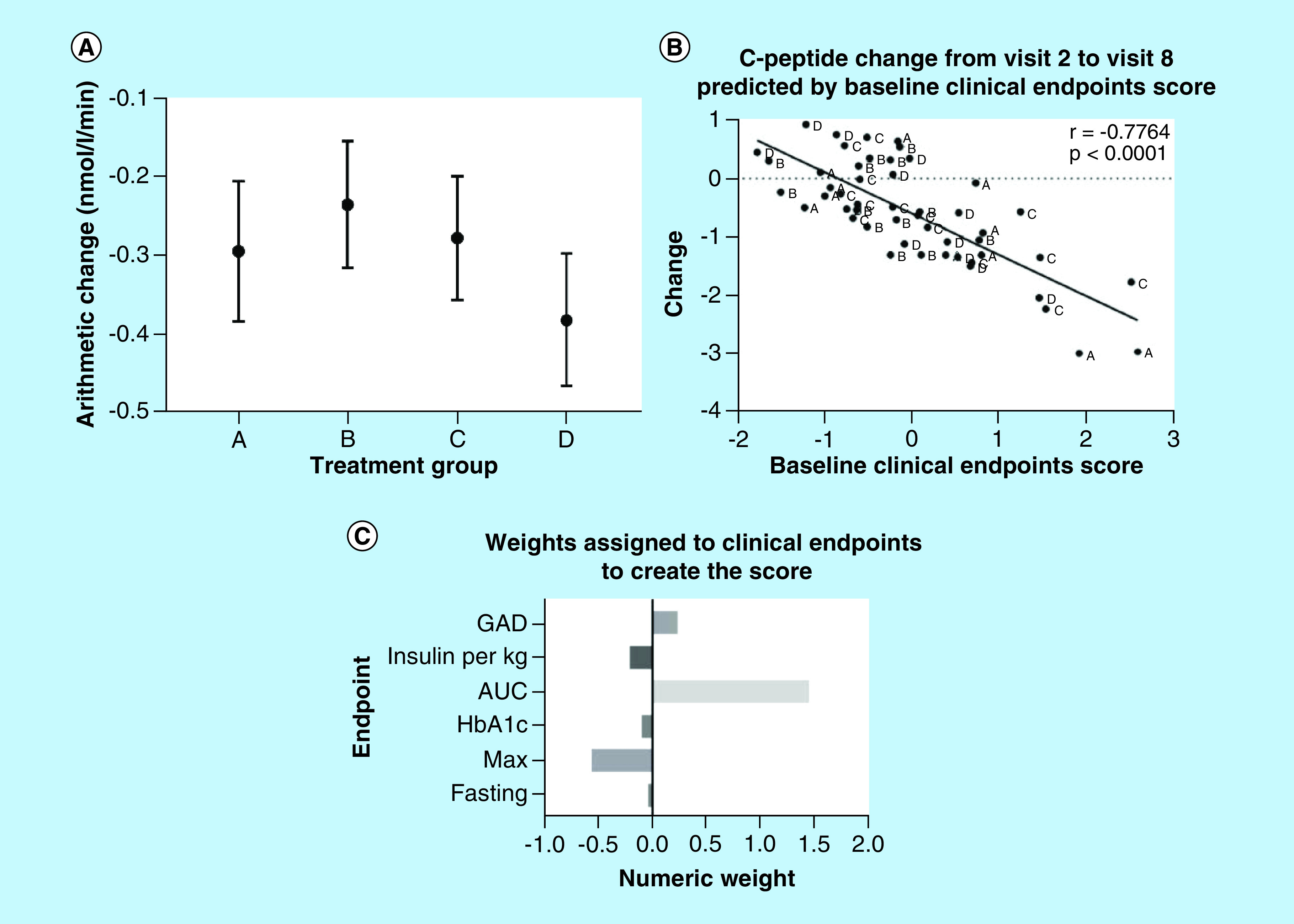
C-peptide response to treatment and C-peptide change predicted by baseline clinical end points. **(A)** Mean (95% CI) arithmetic change in C-peptide AUC/120 from 6 to 30 months-PP population. **(B)** Score values generated by CCA are plotted against arithmetic change in C-peptide AUC/120 from baseline to visit eight (30 months). The correlation between the score and change is estimated to be -0.7764 and statistically significant. **(C)** Weights assigned to each end point by canonical correlation analysis (CCA). Bar chart showing the numeric weights assigned by CCA to each variable and predicting change in C-peptide AUC/120. GAD, baseline AUC/120 and maximum baseline C-peptide are most prominent in determining the clinical score. The score increases with increases in GAD and AUC while decreasing with the other variables.

As seen in Table 1, there were some differences between the patients in the different groups at baseline with, for example, shorter duration of diabetes at inclusion in group D, with somewhat lower fasting blood glucose and higher median C-peptide AUC. There were also pronounced differences in baseline immunological picture (see below). In an effort to overcome the influence of baseline differences between the treatment arms, a post hoc examination of changes from 6 months (considered the ‘post-remission’ phase) to 30 months was performed (Supplementary Table 1). That analysis showed that the combination of GAD-alum (Diamyd^®^) and vitamin D (Group B and C) reduced the slope of decline compared with placebo (p < 00.05; Figure 2A) even though the placebo group D still had slightly higher C-peptide at 30 months. The mean increase of HbA1c in those patients was also significantly lowered by 8.6 mmol/mol when compared with the observed increase in the placebo group at 30 months post-baseline.

CCA analysis of the data revealed that some baseline characteristics were quite important for the C-peptide preservation and the slope of decline (Figure 2B & C). Thus, decline of C-peptide AUC from baseline to 30 months was associated with baseline characteristics as GADA levels, insulin dose/kg/24 h, and HbA1c (correlation of end point score with C-peptide change = – 0.7764, p < 0.0001).

**Figure 3.**
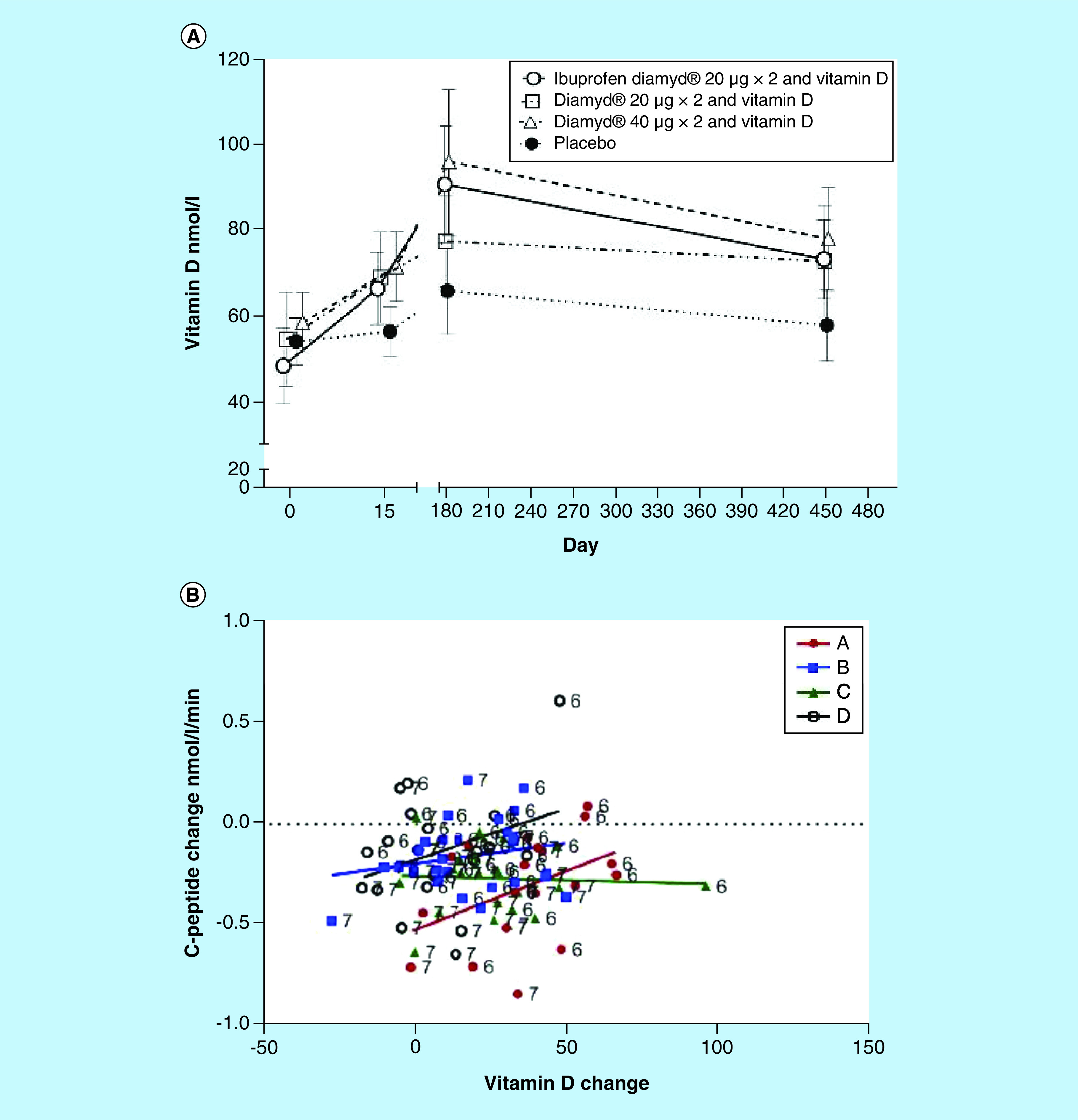
The effect of vitamin D treatment on vitamin D concentrations and association between vitamin D increase and C-peptide. **(A)** Increase of vitamin D concentrations in the three arms treated with 2000 U/day. **(B)** Association between increasing vitamin D from baseline and reduced loss of C-peptide. Arithmetic change in C-peptide AUC/120 min for each subject and from baseline to visit six and visit seven. Treatment group assignments indicated by different colors and alphabetic letter. Trend lines fit within each treatment group are superimposed. Treatment group key: A=Ibuprofen Diamyd^®^ 20 mg×2 and vitamin D, B=Diamyd^®^ 20 mg×2 and vitamin D, C=Diamyd^®^ 40 mg×2 and vitamin D, D = placebo. Significant differences are indicated by p-values.

For vitamin D at baseline, 24 patients had a serum 25-OH vitamin D level below the normal range (<50 nmol/l). While vitamin D levels remained almost stable in the placebo-treated group (D), they increased significantly in the treatment groups (A–C; Figure 3A.). There was a positive but not statistically significant (p = 0.1479) trend between an increase in serum vitamin D and C-peptide levels (Figure 3B).

### Effect of the treatment & the immune response

**Figure 4.**
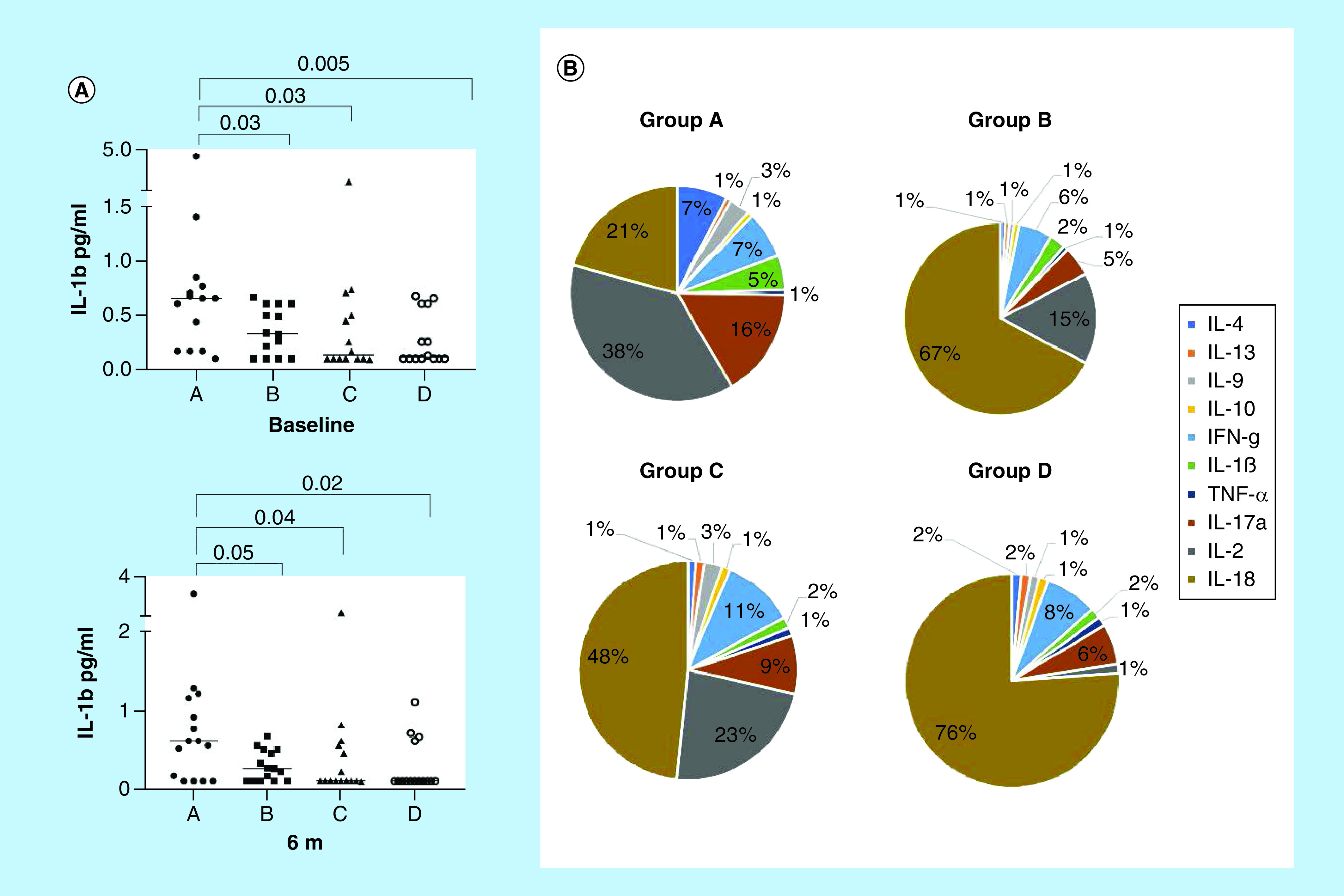
Baseline cytokines levels in serum. **(A)** Median levels (horizontal line) of IL-1 (pg/ml) at baseline and 180 days for A (Ibuprofen + Diamyd^®^ 20 mg×2 + vitamin D; black circles); B (Diamyd^®^ 20 mg×2 + vitamin D, black squares); C (Diamyd^®^ 40 mg×2 + Vitamin D, black triangles); D (Placebo, open circles) were detected by Luminex. **(B)** Relative contribution (%) of the cytokines at baseline. Median values are indicated by horizontal lines. Significant differences are indicated by p-values.

Analysis of baseline cytokine levels in serum showed that IL-1 was higher in group A compared with all the other treatment groups, and remained higher in that group of patients over the study (Figure 4A). Illustration of the cytokine profile revealed predominant proportion of IL-1, IL-2, IL-17 and IFN cytokines in the group A, followed by C, B and D (67, 45, 28 and 17%, respectively (Figure 4B). No differences of chemokine levels in serum was observed, except for the reduction of IL-10 in the samples from the treatment groups B and C along the study, but not in the A and placebo groups (data not shown).

**Figure 5.**
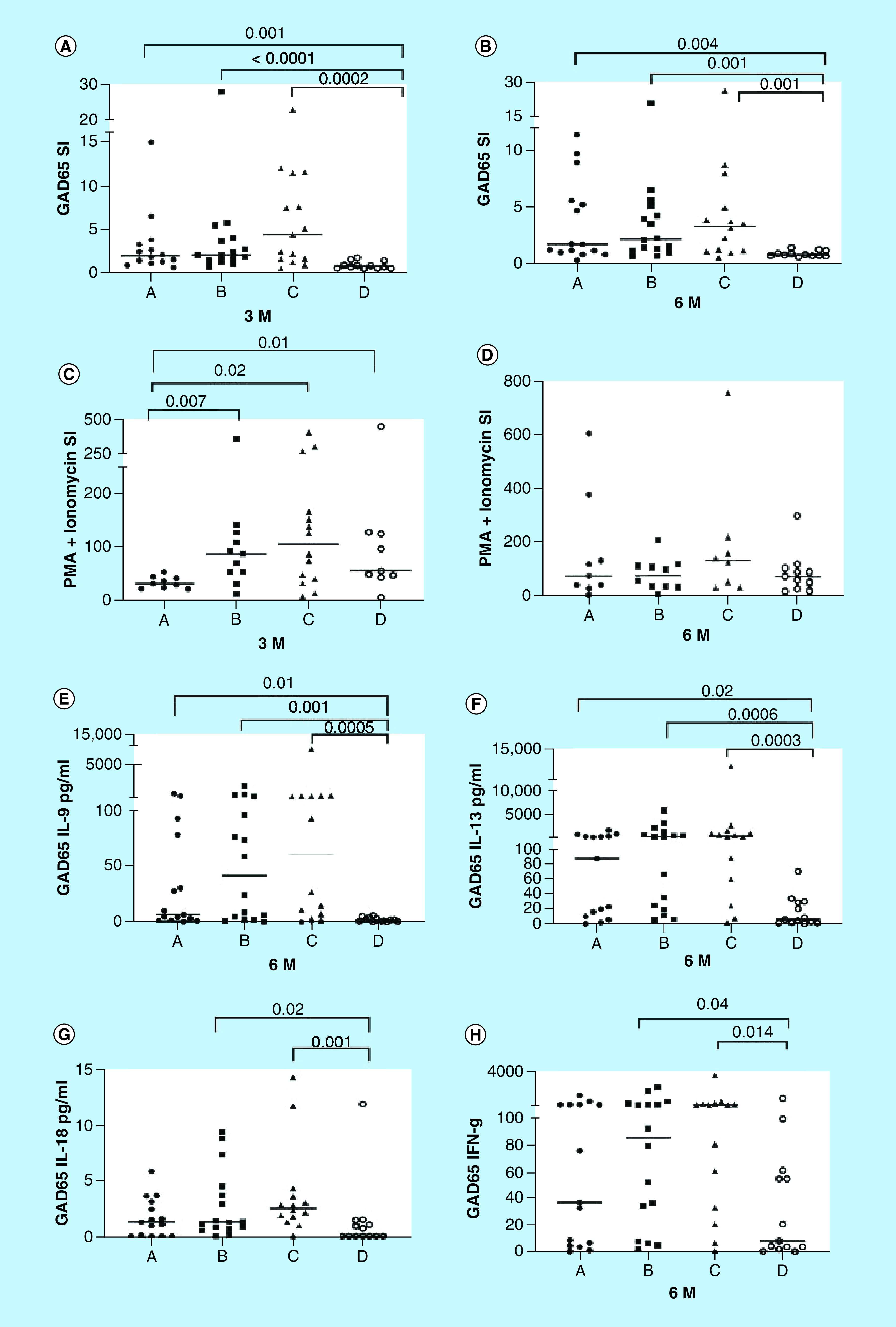
Effect of the treatment and the immune response. Proliferative response to GAD_65_ at **(A)** 3 and **(B)** 6 months. Proliferative response to PMA/Ionomycin at **(C)** 3 and **(D)** 6 months. Proliferation is expressed as stimulation index (SI), calculated from the mean of triplicates divided by the mean of triplicates with medium alone. **(E–H)** GAD_65_-induced cytokine secretion at 6 months upon *in vitro* PMBC stimulation. Cytokines were detected by Luminex in supernatants collected after 7 days culture in presence of medium or GAD_65_ (5 μg/ml). GAD_65_-induced cytokine secretion is given after subtraction of spontaneous secretion from each individual. Median levels (horizontal line) of cytokine (pg/ml) at baseline and 180 days for A (Ibuprofen + Diamyd^®^ 20 mg×2 + Vitamin D; black circles); B (Diamyd^®^ 20 mg×2 + vitamin D, black squares); C (Diamyd^®^ 40 mg×2 + vitamin D, black triangles); D (placebo, open circles). Median values are indicated by horizontal lines. Significant differences are indicated by p-values.

GAD-alum treatment enhanced GADA levels that were higher in all the groups compared with placebo (Supplementary Figure 1). Quantification of the proliferative response to GAD_65_ showed that proliferation was increased at 3 months in all the groups that received GAD-alum compared with placebo (Figure 5A), and remained higher after 6 months (Figure 5B).

Proliferation response to PMA/ionomycin was lower after 3 months in the treatment arm that got Ibuprofen (group A; Figure 5C). The response differed from treatment arm B, a group that received similar treatment but without Ibuprofen. This difference between the groups was not observed at 6 months, in other words, 3 months after Ibuprofen administration ended (Figure 5D).

GAD stimulation induced higher levels of IL-9 and IL-13 in the all the treatment arms that received injections of GAD-alum (Figure 5E & F). Higher levels of GAD-induced IL-18 and IFN were observed in the groups B and C, but not in the A (Figure 5G & H), which might be related to the effect of Ibuprofen on the immune response.

## Discussion

Although hundreds of methods have been shown to stop or delay the onset of autoimmune diabetes in experimental animals, no one has shown good efficacy in humans and therefore further clinical trials in humans are critical. Unfortunately, well-powered double-blind placebo-controlled trials with adequate time for follow-up can take years, and there is not enough collaboration in large networks nor enough resources to organize multiple parallel and/or overlapping clinical trials for substantial progress. Therapies with new approaches and/or using combinations of pilot trials are needed, sometimes even without controls [36]. The results obtained from such pilot studies could then be used to guide the design of future full-scale trials, even though one has to be cautious in conclusions as there is a risk of imbalance between the groups, a problem we actually met in this trial. Thus, patients in group D, the placebo group, had shorter duration of diabetes, somewhat lower fasting C-peptide but higher median C-peptide AUC, which does not allow more definite conclusions. Patients in Group D had also lower levels of IA-2-antibodies, which often are associated with a more rapid decline of C-peptide. Furthermore, it should be noted that Group B and C had reduced slope of C-peptide decline compared with the placebo group (D) in spite of that patients in group C had higher GADA levels at baseline which we found negatively influenced the C-peptide preservation. As another example the proportion of IL-1, IL-2, IL-17 and IFN cytokines was 67% in the group A, but only 17 % in Group D, which might be one possible explanation to the poor response of treatment in group A. This makes it difficult to see any desirable effect of ibuprofen in this group. Furthermore, when trying to elucidate mechanisms using different immunological markers there could be a risk with multiple testing. With our focus on immune response markers such as Th1 resp Th2 deviation and T-cell regulation, based on our earlier GAD-alum studies we tried to minimize these problems, and therefore we did not use correction for multiple testing.

In addition to the above-mentioned observations other results from our pilot trial provided some valuable information. The combination therapy used was safe, the treatment was easy and tolerable for the patients. Next, our data show that the baseline characteristics, both clinical and immunological, seemed to have an impact on the course of the disease and further decline of C-peptide. In addition to the importance of baseline insulin dose, HbA1c and C-peptide, we noticed a greater proportion of IL-1, IL-2, IL-17 and IFN, and higher levels of IL-1β at baseline samples in the group A. Interestingly, IL-1 levels were higher not only at baseline in study arm A, but remained higher along the study in this group. The anti-inflammatory effect of Ibuprofen might had some effect on the immune response, as lower proliferation after stimulation with PMA/Ionomycin was observed in the same group, while GAD-induced inflammatory cytokines were not increased. This might be an interesting outcome, as GAD-alum treatment in absence of Ibuprofen induced a broad range of cytokines, in agreement with previous findings [37,38]. However, the study arm A, receiving Ibuprofen, had the most rapid decline of C-peptide from 6 to 30 months. This is in agreement with previous findings that serum IL-1 correlated negatively with beta-cell function in patients with new-onset Type I diabetes [39]. It has been shown that short-term use of ibuprofen results in a ‘rebound’ increase in cytokine-induced IL-1-β and TNF synthesis [40]. *In vitro* stimulation with Ibuprofen enhanced IL-1β secretion by PBMCs in schizophrenic patients but not in healthy individuals [41]. Thus, it cannot be excluded that short-term use of ibuprofen may even worsen immune-mediated β-cell destruction by increasing IL-1 levels, as it has been previously demonstrated in other settings. Thus a lesson from our trial is that baseline data should be considered not only when designing studies, but also when evaluating the effect of different treatments, as a certain treatment regimen may be efficacious in a subgroup of patients but fail when simply estimating the efficacy in a general Type I diabetes population. Our study underlines the importance of considering Type I diabetes heterogeneity especially when designing clinical trials [42]. In addition, it is important to be aware that the immune system dictates outcomes of immunotherapies [43] which is relevant also in the context of GAD-alum studies [44]. We need to learn from pediatric oncology to stepwise improve efficacy by better analyzing which subgroups of patients respond to a certain treatment, perhaps a combination therapy, while others need other variants of therapy.

The addition of vitamin D 2000 IU/day did lead to a measurable increase of 25-hydroxy-vitamin D levels in the serum. When vitamin D was used in combination with GAD-alum twice at monthly intervals without the combination with Ibuprofen, those patients had a tendency to less rapid decline of C-peptide from 6 to 30 months compared with patients in the placebo groups. The decline of C-peptide was related to baseline serum vitamin D, and an increasing serum vitamin D level was positively correlated with C-peptide preservation, although not significantly. This is an interesting finding when looking at the results of previous GAD-alum studies. For instance, in the Phase II trial showing efficacy of the treatment [14] all patients were treated in the spring with an impressive efficacy. In the following Phase III trial [16] there was significant efficacy in the subgroup treated during the spring, when vitamin D levels are supposed to be increasing due to sun exposure. Even though all treatment arms in the actual study failed to reach end point for C-peptide preservation, our results may suggest that increase of vitamin D in serum, with effects on the immune regulation, may improve efficacy of auto-antigen treatment. It might even be so that higher vitamin D concentrations might have given even better efficacy [45]. Most important is to reach an adequate serum concentration [46].

Previous studies have indicated that the GAD-alum dose should be somewhere between 20 and 100 μg, and therefore we used two different doses in this trial. The higher dose 40 μg×2 was not convincingly better than giving GAD-alum 20 μg×2.

As baseline clinical and immunological characteristics differed between the treatment arms, in order to see if using data after the well-known ‘honeymoon period’ would be as or more informative than the conventional baseline, we investigated the use of C-peptide data collected at 6 months of treatment as baseline. We chose 6 months even though we are aware of that duration of partial remission varies in different studies [47,48]. Our findings suggest that this strategy might possibly be informative to estimate the efficacy diminishing the consequence of baseline situation and should be further explored with larger number of patients. Our data will be available when there is relevant scientific study ethically approved and our data can be of value to improve knowledge and science.

In summary, this pilot trial illustrates that even a small under-powered study can provide valuable information for the design of larger trials. The efficacy of GAD-alum treatment to preserve β-cell function seemed to be influenced by baseline serum vitamin D levels, and increasing serum vitamin D levels may be associated with C-peptide preservation. It is reasonable to include vitamin D in further studies of GAD-alum trials with the aim to reach relevant serum concentrations, but we find no real support for further use of Ibuprofen, which, beside some immunological effects, did not contribute to β-cell preservation. Most importantly, baseline clinical and immunological data should be considered in the design and evaluation of future immune interventions.

## Future perspective

Even with the most modern devices for glucose control the aim must be to cure Type I diabetes. One way to step-wise reach this goal is to learn how to preserve and improve residual β-cell function. As Type I diabetes is heterogenous, we need to learn how to select the right patients for adequate therapies based on both genetic, clinical and immunological characteristics. In some cases, immune suppression may be efficaceous, but it will be difficult to avoid unacceptable adverse events and risks. Auto-antigen treatment may become a safe way forward but we need to know more about the route of administration, doses, what autoantigens to use in different patients [49]. Furthermore, we will probably need to combine different regimens and agents, in a similar way as been used with great success in, for example, pediatric oncology [34]. Our study supports the addition of vitamin D, but more clinical studies will tell us how to make different combination therapies with different auto-antigens, immune modulation agents and protective agents.

**Summary points**

- Type I diabetes is the most common life-threatening disease in children and adolscents in many countries. There is no prevention and no cure. Preservation of residual β-cell function makes Type I diabetes milder and is a step toward cure. So far existing immune interventions have not been enough efficaceous, are heavy for the patients with adverse events and sometimes unacceptable risks.
- Combination therapies including autoantigen treatment to stop the autoimmune process is a promising approach. Glutamic acid decarboxylase-alum has shown efficacy in certain groups, but we have to learn how to make such treatment more effective.
- Clinical studies are needed, and pilot trials may give valuable information. We have tried a a combination of autoantigen treatment with glutamic acid decarboxylase-alum sc, vitamin D and anti-inflammatory treatment with Ibuprofen in children with recent onset Type I diabetes. This intervention was safe and tolerable, easy for the patients and healthcare.
- Type I diabetes is heterogenous and our results underline the importance of basal clinical and immunological picture for treatment results. Vitamin D supplementation to reach adequate serum concentrations seems to be helpful, while a short Ibuprofen treatment shows no benefit.
